# To be or not to be associated: power study of four statistical modeling approaches to identify parasite associations in cross-sectional studies

**DOI:** 10.3389/fcimb.2014.00062

**Published:** 2014-05-15

**Authors:** Elise Vaumourin, Gwenaël Vourc'h, Sandra Telfer, Xavier Lambin, Diaeldin Salih, Ulrike Seitzer, Serge Morand, Nathalie Charbonnel, Muriel Vayssier-Taussat, Patrick Gasqui

**Affiliations:** ^1^INRA, UR346 Epidémiologie AnimaleSaint Genès Champanelle, France; ^2^INRA-Anses-ENVA, USC BIPARMaisons-Alfort, France; ^3^School of Biological Sciences, University of AberdeenAberdeen, UK; ^4^Department of Ticks and Tick-borne Diseases, Veterinary Research InstituteKhartoum, Sudan; ^5^Division of Veterinary-Infection Biology and Immunology, Research Center BorstelBorstel, Germany; ^6^Institut des Sciences de l'Evolution (CNRS /IRD / UM2), University of Montpellier 2Montpellier, France; ^7^Animal et Gestion Intégrée des Risques, CIRADMontpellier, France; ^8^INRA, UMR CBGP (INRA / IRD / CIRAD / Montpellier SupAgro)Montpellier, France

**Keywords:** associations, interactions, modeling, parasite community, screening, GLM approach, network model, chi-square test

## Abstract

A growing number of studies are reporting simultaneous infections by parasites in many different hosts. The detection of whether these parasites are significantly associated is important in medicine and epidemiology. Numerous approaches to detect associations are available, but only a few provide statistical tests. Furthermore, they generally test for an overall detection of association and do not identify which parasite is associated with which other one. Here, we developed a new approach, the association screening approach, to detect the overall and the detail of multi-parasite associations. We studied the power of this new approach and of three other known ones (i.e., the generalized chi-square, the network and the multinomial GLM approaches) to identify parasite associations either due to parasite interactions or to confounding factors. We applied these four approaches to detect associations within two populations of multi-infected hosts: (1) rodents infected with *Bartonella* sp., *Babesia microti* and *Anaplasma phagocytophilum* and (2) bovine population infected with *Theileria* sp. and *Babesia* sp. We found that the best power is obtained with the screening model and the generalized chi-square test. The differentiation between associations, which are due to confounding factors and parasite interactions was not possible. The screening approach significantly identified associations between *Bartonella doshiae* and *B. microti*, and between *T. parva*, *T. mutans*, and *T. velifera*. Thus, the screening approach was relevant to test the overall presence of parasite associations and identify the parasite combinations that are significantly over- or under-represented. Unraveling whether the associations are due to real biological interactions or confounding factors should be further investigated. Nevertheless, in the age of genomics and the advent of new technologies, it is a considerable asset to speed up researches focusing on the mechanisms driving interactions between parasites.

## Introduction

A growing number of studies of many mammal hosts, including wild and domestic animals and humans, are reporting simultaneous infections by different microparasites (Cox, 2001; Palacios et al., 2009; Saisongkorh et al., 2009; Tadin et al., 2012; Jacquot et al., 2014), macroparasites (Byrne et al., 2003; Behnke, 2009; Fenton et al., 2010) and both (Jolles et al., 2008; Ezenwa and Jolles, 2011; Nunn et al., 2014). The frequency of co-occurrence can be influenced by interactions between parasites. These interactions are of crucial medical concern because they can alter host susceptibility, infection length and clinical symptoms, as illustrated by the influence of helminths on malaria severity (Nacher, 2002). From an epidemiological point of view, interactions can alter the risk of transmission. Parasites can interact in a synergistic manner when the presence of one favors the infection by a subsequent parasite, as, for example, HIV and *Mycobacterium tuberculosis* (Corbett et al., 2003). Parasites can also interact in an antagonistic manner, as, for example, in *Aedes aegypti* mosquitoes, where infection with the symbiotic *Wolbachia* prevents subsequent infection with dengue virus, Chikungunya virus and the agent of malaria (Moreira et al., 2009). Parasite interactions have mostly been considered as a one-to-one interaction, where the infection of one parasite influences the acquisition of and/or dynamics of infection by a second parasite. However, interactions between a set of parasites are conceivable where different parasites interact within a network or through “cascade consequence” (Rigaud et al., 2010; Bordes and Morand, 2011). For instance, such networks have been successfully used to identify interactions in ecology, e.g., El Niño (Trenberth and Fasullo, 2011), in genetics, e.g., HLA genes (Wansen et al., 1997), or in metabolic pathways, e.g., metabolic regulation (Matsuoka and Shimizu, 2012).

The co-occurrence of parasites can also result from confounding factors that create statistical associations between parasites, even though there are no true biological interactions. For instance, similarities in host environment, behavior or susceptibility can cause correlations in the risk of infection between two parasites (e.g., association filters, Combes, 2001). For example, associations in humans between the agent of malaria and helminth infections may be due, in certain contexts, to common social or environmental factors, which can be depicted by a social network analysis, rather than a true biological interaction (Mwangi et al., 2006). Thus, in host populations, interactions between two parasites are suspected when the probability of coinfection is not random once confounding factors have been taken into account.

In populational studies, longitudinal or time series data are useful for identifying parasite associations. However, such studies are resource-intensive. In such studies, one can test whether the presence of a parasite impacts the probability of infection by another one (e.g., Mahiane et al., 2010; Sherlock et al., 2013) or one can test whether the infection dynamics of several parasites are correlated (Rohani et al., 2003). One-off cross-sectional studies are widely used to screen for the presence of several parasites because they are less time and money consuming than longitudinal studies. This is especially the case when emerging or poorly known parasites or host species are studied. Numerous approaches are available to detect parasite associations in such contexts. Although a previous study has assessed different approaches to detecting interactions between macroparasites (Fenton et al., 2010), no study has compared the quality (i.e. the probability to wrongly identify association or the power to detect any association) of different approaches for the categorical data (infected, not infected) that is usually available for parasites.

Multivariate analyses (PCA, FCA, DA, CoA) (Gauch, 1982) evaluate which parasites tend to group together. The drawback is that there is usually no statistical test associated with these analyses (but see for example permutation methods, Tollenaere et al., 2008; Salvador et al., 2011). This is an important drawback because statistical tests determine whether the observations depart or not from the null hypothesis (i.e., the fact that the parasites are independent, i.e., not associated). The chi-square test is the most popular and easy test to implement. It is mainly used to study two parasites, but several adaptations have been proposed to study multiple parasites (Holm, 1979; Benjamini and Hochberg, 1995; Janovy et al., 1995). In particular, the chi-square test can be generalized based on the correlation between two qualitative variables, as described by Fahrmeir and Tutz (1994). The main drawback of this approach is that at least five individuals are required per infectious status. Generalized linear models (GLM) are also widely used with multinomial logistic regression (McCullagh and Nelder, 1989; Yee and Wild, 1996; Agresti, 2002). Such approaches can explicitly account for potential confounding factors. Networks have been increasingly used (Bascompte, 2007) in the last few years in many fields, e.g., in medicine: metabolic pathways (Ravasz et al., 2002; Qin et al., 2012), in computer science: peer to peer networks (Fox, 2001) or in social science: scientific collaboration (Newman, 2004). They also offer an attractive representation of multiple parasite relationships. They provide indices of association such as connectance (Yodzis, 1980), nestedness (Bascompte et al., 2003) or betweeness (Freeman, 1977). However, to date, statistical tests regarding the connectance have rarely been used. All of these approaches have the potential to statistically identify whether there are overall parasite associations within a dataset. However, none statistically identify the specific parasites that are associated. To address this issue, we developed a new approach to study parasite associations, which we called association screening. This approach has the advantage, compared to the others, to detect and statistically test which parasites potentially interact. It is an important pre-requisite for further more precise research focusing on the mechanisms driving any identified interactions, with, for instance, a mechanistic model (e.g., Sherlock et al., 2013; Vaumourin et al., 2013).

The objectives of our study were threefold: (1) to develop a new approach—“association screening”—to statistically test the overall and specific parasite associations within hosts; (2) to compare the “quality” of the new approach and three other known approaches to identify parasite associations in cross-sectional studies, namely the generalized chi-square test, the network model and the multinomial GLM approach. To do so, we developed a statistical test for the networking and the generalized chi-square approach. Using simulations, we verified that the α risk (i.e., the probability to wrongly detect associations) was well controlled *a priori*. Then, still using simulations, we compared the power (i.e., the probability to detect existing associations) of the four statistical modeling approaches, to identify parasite associations either due to parasite interactions or to confounding factors; (3) to apply these four approaches to detect associations using two datasets of multi-infected hosts. The first one was a population of field voles (*Microtus agrestis*) infected with blood parasites (*Babesia microti*, *Anaplasma phagocytophilum* and *Bartonella* sp.) (Telfer et al., 2007, 2010). Potential associations between parasites have already been identified for a longitudinal dataset on *M. agrestis* infections obtained from the same study area (Telfer et al., 2010; Sherlock et al., 2013). Here we used an independent cross-sectional dataset (Telfer et al., 2007). Thus, we were able to discuss whether our results were consistent with what was previously found. The second dataset was used to screen associations in a population of bovine infected with *Theileria* sp. and *Babesia* sp. (Salih et al., 2007). To our knowledge, such associations have never been investigated, despite the observations of co-occurrence within vectors (e.g., Ica et al., 2007; ìa-Sanmartı, ìn, Barandika, Juste, Garcı) and hosts (e.g., Nagore et al., 2004; Altay et al., 2008). Our results should thus underline possible interactions between those parasites.

## Materials and methods

### Four statistical modeling approaches

#### Overall modeling implementation

For each of the models except the generalized chi-square, we constructed a statistical test, which is based on a simulated theoretical distribution of a statistic and its associated confidence interval under the null hypothesis H0 that parasite associations are random.

For each model, to estimate the simulated statistic distribution, we simulated NS (Number of Simulations) instances (with NS ≥ 1000) of a dataset with the same number of parasites (NP), the same observed probability of each i parasite (*p*_*i*_) and the same total number of hosts (NH). For a given NP, the number of possible parasite combinations NC was calculated as NC = 2^NP^. Parasite combinations are exclusive of one another. The occurrence probability of each NC combination of parasites (Q vector) was calculated as function of {*p*_*i*_, 1 ≤ *j* ≤ NP}. Under H0, we have *Q* = {*q*_*j*_, 1 ≤ *j* ≤ NC} with $q_{j}=\prod_{i\;=\;1}^{i\;=\;NP}\left({\left(p_{i}\right)}^{1_{i}^{j}}.{\left(1-p_{i}\right)}^{1\;-\;1_{i}^{j}}\right)$ and indicator 1^*j*^_*i*_ = 1 if a parasite i was present in a j combination, or 0 otherwise. In each dataset and for each host, under the null hypothesis H0, an i parasite is associated at random with a Bernoulli probability distribution with parameter p_*i*_, or a parasite combination is associated at random with a multinomial probability distribution with Q parameter vector. The statistics is evaluated for each simulated dataset. A 95% confidence interval was estimated using the distribution of all simulated statistics.

Similarly, for the observed statistics, the decision criteria (i.e., rejection or not of H0) and the associated *p*-value for each of the developed models were obtained with the simulated statistic 95% confidence interval. This method is similar to the bootstrap technique (Efron, 1979; Davison et al., 1986).

All programs used in the analysis were written using R software (version 3.0.1) accessible on the site http://cran.r-project.org/ (see the name of the packages used below).

#### Association screening approach

The association screening approach is based on the statistic distribution of the occurrence count of each possible combination of parasites under H0. A simulated dataset was an absence/presence matrix with hosts in lines and parasite combinations in columns.

With NS = 5000 simulations, we obtained the NC statistic distributions. We estimated a 95% confidence envelope to obtain a profile that includes simultaneously all the combinations. From this profile, we inferred for each combination two quantiles, *Q*^*inf*^_*j*_ and *Q*^*sup*^_*j*_, as $P\left\{\cap_{j\;=\;1}^{j\;=\;NC}\left\{Y_{j}\in \left[Q_{j}^{inf};Q_{j}^{sup}\right]\right\}\right\}=0.95$. A global test was based on the 95% confidence envelope. When H0 was rejected, the local tests were based on the NC confidence intervals: $\left[Q_{j}^{inf};Q_{j}^{sup}\right]$.

We used the *envelope* function from the *boot* package to estimate a 95% confidence envelope for the combination count distribution profile (Davison, 1997) (for more details on implementation see Script 1).

#### Generalized chi-square approach

The generalized chi-square approach is based on the chi-square distribution, without any simulation step. An observed dataset was an absence/presence matrix with hosts in lines and parasite combinations in columns.

If *Yobs*_*j*_ was the number of hosts observed with the parasite combination j, the statistic [CHI2] under H0 was defined by: $CHI2=\sum_{j=1}^{j\;=\;NC_{5}}{\left(Yobs_{j}-q_{j}.NH\right)}^{2}/\left(q_{j}.NH\right)+\left(\text{​}{\left(\left(\sum_{j\;=\;NC_{5}}^{j\;=\;NC}Yobs_{j}\right)-\left(NH.\sum_{j\;=\;NC_{5}}^{j\;=\;NC}q_{j}\right)\right)}^{2}\text{​}/\left(NH.\sum_{j\;=\;NC_{5}}^{j\;=\;NC}q_{j}\right)\text{​}\right),$ with NC_5_ the number of combinations where the number of host individual (*q*_*j*_*.NH*) was superior or equal to 5 (Agresti, 2002). Therefore, the combinations, where the number of individuals was inferior to 5, were merged together. With the generalized chi-square distribution for NC_5_ degrees of freedom, the 95% confidence interval was obtained. In the event of rejection of H0, it is possible to isolate the combination of parasites which contributed the most to the statistic CHI2 [i.e., with a greater contribution to generalized chi-square than the mean value (χ^2^_*obs*_/*NC*)], without this amounting to a real local test (for more details on implementation see Script 2).

#### Multinomial GLM approach

The multinomial GLM approach is based on the statistic distribution of the residual deviance under H0, obtained with a GLM model and a multinomial family (McCullagh and Nelder, 1989). A simulated dataset was an absence/presence matrix with hosts in lines and parasite combinations in columns.

*Y*_*j*_ was the number of hosts with the parasite combination j, the residual deviance under H0 was defined by: *resDev* = −2. $resDev=-2.\sum_{j=1}^{j=NC_{1}}Y_{j}.\text{log}(Y_{j}/NH)$, with NC_1_ the number of combinations like *Y*_*j*_ not equal to 0. The number of degrees of freedom associated was defined by: *resddl* = (*NC*_1_ − 1). (*NH* − 1). In the GLM approach, the statistic [GLMC] was defined by: *GLMC* = *resDev/resddl*. With NS = 1000 simulations, we obtained the statistic distribution, and the 95% confidence interval was estimated with the quantiles *Q*^0.025^ and *Q*^0.975^.

We used the *vglm* function from the *VGAM* package to realize a multinomial logistic regression model (Yee and Wild, 1996) (for more details on implementation see Script 3).

#### Network approach

The network approach is based on the statistic distribution of the network connectance under H0. The connectance is a structural index (comprised between 0 and 1), which represents the proportion of observed links relative to the number of possible links. It describes the overall complexity of the network (Wasserman and Faust, 1994).

For a given dataset two connectance results were obtained: the network connectance for host individuals in relation to parasites [PNWC] (i.e., two hosts were connected if they shared the same parasite) or in relation to parasite combinations [CNWC] (i.e., two hosts were connected if they shared the same parasite combination). A simulated dataset was an absence/presence matrix with hosts in lines and parasites or parasite combinations in columns. We chose to work on both, parasite and combination networks because parasite networks are usually performed whereas combination network is directly comparable to the other approaches studied here.

In the case of parasite combinations, with *Y*_*j*_ the host number with the parasite combination j, the combination network connectance statistics was defined by: $CNWC=\sum_{j=1}^{j=NC}\left(\left(Y_{j}\cdot \left(Y_{j}-1\right)\right)/\left(NH\cdot \left(NH-1\right)\right)\right)$. The parasite network connectance statistics was defined by: $PNWC=\sum_{k=2}^{k=NH}\left(\sum_{l=1}^{l=k-1}\left(1_{l}^{k}/\left(NH\cdot \left(NH-1\right)/2\right)\right)\right)$, with indicator 1^*k*^_l_ = 1 if the host individuals k and l had at least one common parasite, or 0 otherwise. With NS = 1000 simulations, we obtained two statistic distributions, and the two 95% confidence interval estimated with, in each case, the quantiles *Q*^0.025^ and *Q*^0.975^.

Graphical representations of the structuring of hosts and parasites were realized using respectively the projections of hosts and parasites of a parasite network approach. However we did not study the associated statistics connectance because it is not informative given the low number of parasites.

We used the *igraph* package, in particular the *graph.density* connectance function (Csárdi and Nepusz, 2006) (for more details on implementation see Script 4).

### Sensitivity studies of the modeling approaches

For each model, we first checked whether the α risk, i.e., the risk to conclude that the association (alternative hypothesis H1) is significant when in fact the association was random (null hypothesis H0), defined *a priori*, was close to 0.05.

Next, we performed NS simulations (NS = 1000) of a dataset under the null hypothesis H0. The estimated **α** was the frequency of rejecting H0 using these new NS simulations.

To perform these sensitivity studies, we considered that the parasites were not associated, i.e., H0 hypothesis. The population size was 1000 hosts. Below, we also studied a smaller population of 500 individual hosts for the power studies. 1000 and 500 are reasonable numbers of hosts to have in a field study. The number of parasites varied from two to eight, which gave 256 combinations (i.e., <500 hosts). We did not study higher numbers of parasites because we need to keep the number of combinations below that of hosts. Parasite prevalences were uniformly distributed between 0.10 and 0.60, this interval was wide enough to give a trend. We performed the simulations for 7 levels of NP (i.e., 7 levels of the simulation scheme as NH was held at 1000).

#### Power studies of the modeling approaches

For each model we evaluated the **β** risk, corresponding to the probability of concluding that the association was random (H0) when there was association (H1). Then, the power was evaluated by (1 − β) which is the capacity to accept H1 under hypothesis H1.

For each model, we performed NS simulations (NS = 1000) of a dataset under the null hypothesis H1. The estimated (1 − β) was the frequency with which H0 was rejected with the new NS simulations.

The ideal approach to detect biological parasite interactions would be an approach that would be able to detect parasite association, except in the case of confounding factors. We considered here that confounding factors structure host populations into subpopulations that have different levels of parasite prevalence. To mimic this, we created subpopulations that have different sizes and parasite prevalence. We independently generated parasite occurrence. Therefore, there was no parasite association within each subpopulation. Thus, we constructed two H1 hypotheses. The first one is that host populations are structured (H1h) but not parasites (H0p). The second H1 hypothesis is that parasites are biologically associated (H1p) and host populations are not structured (H0h). Thus, we checked whether different statistics were specific to each hypothesis, i.e., powerful statistics to detect host population structure only, and other statistics that detect parasite structure only.

In all cases, we studied two sizes of the general host population (i.e., a large population NH = 1000 or a small population NH = 500) and up to eight parasites (i.e., 256 combinations). Parasite prevalences were uniformly distributed between 0.10 and 0.60.

In the case of the hypothesis of host structuring (H1h) without parasite structuring (H0p), host subpopulations differed both by their sizes and by the prevalences of their parasites. Four sets of two host subpopulations were tested, with the ratio between the sizes of the two subpopulations set at 0.2 (i.e., strong imbalance in size between the two subpopulations, e.g., for a general population where NH = 1000, subpopulation 1: NH_1_ = 200 and subpopulation 2: NH_2_ = 800), 0.3, 0.4 or 0.5 (i.e., balance in size between the two subpopulations, e.g., for a general population where NH = 1000, subpopulation 1: NH_1_ = 500 and subpopulation 2: NH_2_ = 500). The parasite prevalence ratio within the first subpopulation was either 0.5 or 1.5 times the prevalence found under H0h. Parasite prevalence within the second subpopulation is defined so that the overall parasite prevalence is equal to those defined under H0h. Thus, the parasite prevalences within the first subpopulation were respectively lower or higher than the second subpopulation, e.g., for a general population where the prevalence of parasite 1 was 0.10%, it was, for the subpopulation 1, respectively 0.05 or 0.15%, and the opposite for the subpopulation 2. We obtained 112 levels for the simulation scheme, i.e., 2 levels of NH ^*^ 7 levels of NP ^*^ 2 levels of parasite prevalence ratios ^*^ 4 levels of subpopulation size ratios.

In the case of the hypothesis parasite structuring (H1p) without host structuring (H0h), we defined four correlation levels between the two parasites that had the two highest prevalences, so that the overall parasite prevalences are the same as under H0p hypothesis. The other parasites were not correlated. The correlation between the two parasites of prevalence p_1_ and p_2_ under H0p, was defined (Fahrmeir and Tutz, 1994) by: *cor*(*y*_1_, *y*_2_) = [*P*(*y*_1_ = *y*_2_ = 1) − *p*_1_ · *p*_2_]/[*p*_1_ · (1 − *p*_1_) · *p*_2_ · (1 − *p*_2_)]^1/2^. Thus, to obtain the prevalence p_1_ and p_2_ under H0p, the studied correlations were fixed to four values: *min*(*cor*), *min*(*cor*)/2, *max*(*cor*)/2 and *max*(*cor*), with *min*(*cor*) = *max*(−1; *a*; 1/*a*) and *max*(*cor*) = *min*(1; *b*; *c*; ((1/*d*) − (1/*a*))). The constraints a, b, c and d were function to p_1_ and p_2_: a = [*p*_1_ · *p*_2_/((1 − *p*_1_) · (1 − *p*_2_))]^1/2^, b = [*p*_2_ · (1 − *p*_1_)/(*p*_1_ · (1 − *p*_2_))]^1/2^, c = [*p*_1_ · (1 − *p*_2_)/(*p*_2_ · (1 − *p*_1_))]^1/2^ and d = [*p*_1_ · (1 − *p*_1_) · *p*_2_ · (1 − *p*_2_)]^1/2^. We obtained 56 levels for the simulation scheme, i.e., 2 levels of NH ^*^ 7 levels of NP ^*^ 4 levels of correlations.

#### Case studies: association studies in two populations infected by multiple parasites

We applied these four models to detect associations within two datasets of multi-infected hosts (Salih et al., 2007; Telfer et al., 2007, 2010). The first dataset was composed of 887 field voles (*Microtus agrestis*), sampled from 27 sites in the United Kingdom biannually between 2001 and 2004. Among field voles, 23 were infected by BGA (an unidentified *Bartonella* species) (2.6%), 137 by *B. doshiae* (15.4%), 105 by *B. grahamii* (11.8%), 186 by *B. taylorii* (20.9%), 305 by *Babesia microti* (34.4%) and 36 by *Anaplasma phagocytophilum* (4.1%) (for more information on the sampling and *Bartonella* diagnostic assays see Telfer et al., 2007; for more information on the diagnostics for *B. microti* and *A. phagocytophilum* see Telfer et al., 2010).

The second dataset was composed of 600 cattle (local Zebu, long-horned Nilotic type of cattle), sampled in Sudan in 2005. Among them, 427 were infected by *Theileria parva* (71%), 436 by *T. mutans* (73%), 272 by *T. velifera* (45%), 16 by *T. taurotragi* (2.7%), 3 by *T. buffeli* (0.5%), 1 by *T*. *annulata* (0.2%), 10 by *Babesia bovis* (1.7%) and 2 by *B. bigemina* (0.3%) (for more information see Salih et al., 2007).

## Results

### Sensitivity studies of the modeling approaches

**Figure 1:**
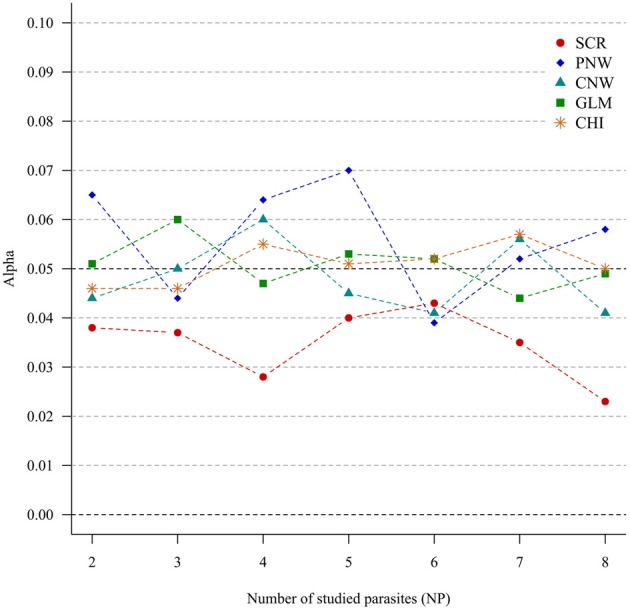
**Simulation outputs relative to the test of the sensitivity (α) for the four approaches**. CHI, generalized chi-square test; PNW, parasite network model; CNW, combination network model; GLM, multinomial GLM model and SCR, the association screening approach. The number of parasites varied from two to eight, the size of the total host population was fixed to 1000.

For all approaches, α risk had equal (generalized chi-square test, network approaches and multinomial GLM model) or lower (association screening model) to 0.05% as expected (Figure 1).

### Power studies of the modeling approaches

#### Host structuring (H1h) without parasite structuring (H0p)

**Figure 2:**
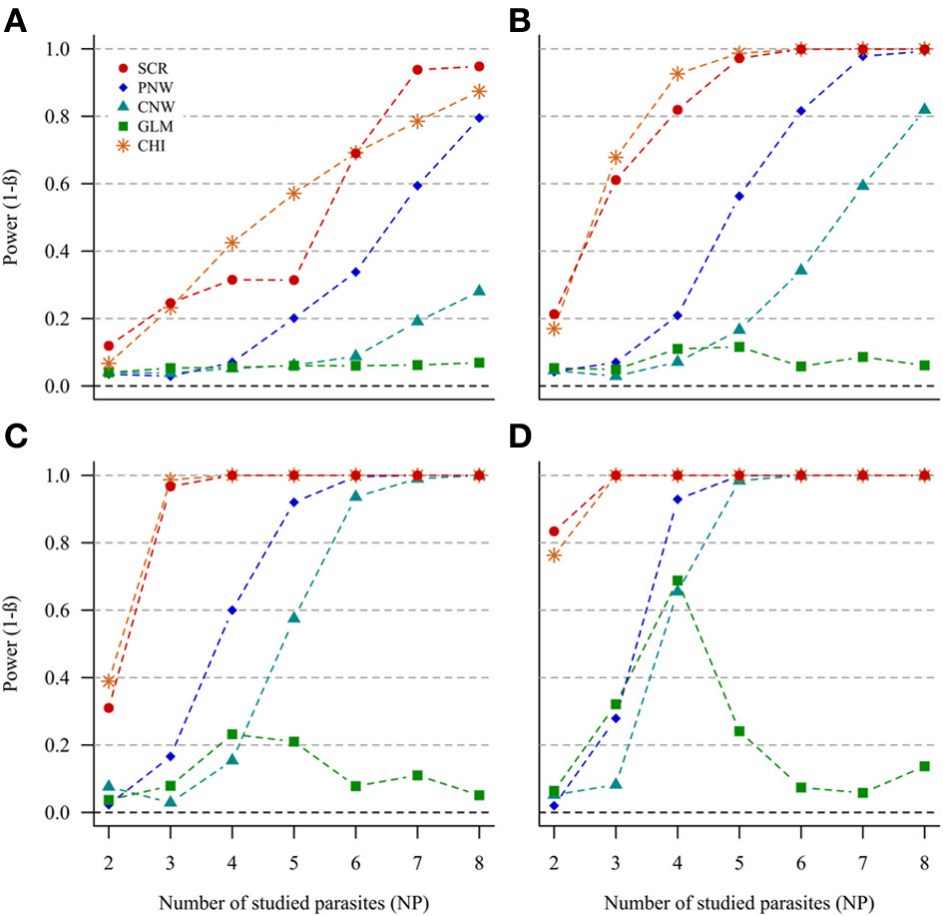
**Simulation outputs relative to the test of power (1-β) for the four approaches used to detect host structuring, with a prevalence ratio equal to 0.5**. CHI, generalized chi-square test; PNW, parasite network model; CNW, combination network model; GLM, multinomial GLM model and SCR, the association screening approach. The number of parasites varied from two to eight, the size of the total host population was fixed to 1000 and the ratios of host subpopulation sizes varied **(A)** 0.2. **(B)** 0.3. **(C)** 0.4. **(D)** 0.5.

In the case of NH = 1000 and for both ratios of prevalence levels, the two best models were the association screening and the generalized chi-square (Figure 2, Annex 1). On the contrary, the least powerful approach, whatever conditions, was the GLM model (Figure 2). For all approaches, except the GLM, we observed that the power increased with increasing number of parasites. Thus, our ability to reveal a subpopulation structure in the case where only two parasites are studied was very poor. The power was higher when the number of host individuals in the two subpopulations was similar.

The same trends were observed for the smaller host population size (NH = 500), but power was moderately lower (see Annexes 2 and 3).

#### Parasite structuring (H1p) without host structuring (H0h)

**Figure 3:**
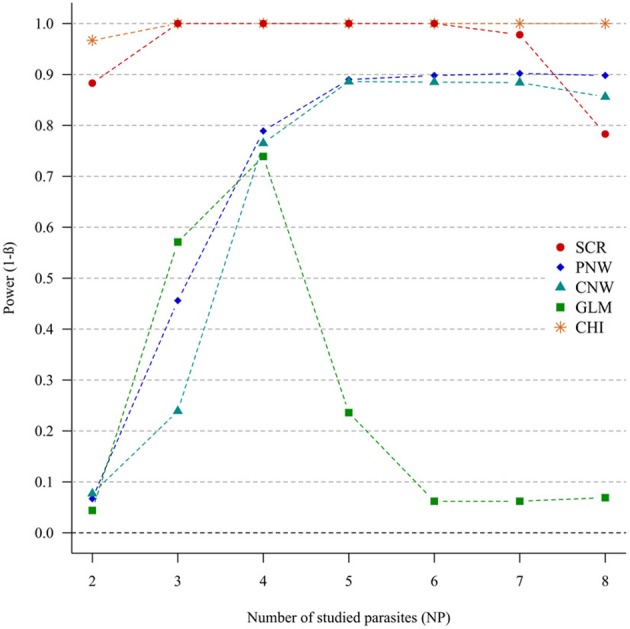
**Simulation outputs relative to the test of power (1-β) for the four approaches used to detect parasite structuring, with weak positive correlations**. CHI, generalized chi-square test; PNW, parasite network model; CNW, combination network model; GLM, multinomial GLM model and SCR, the association screening approach. The number of parasites varied from two to eight and the size of the total host population was fixed to 1000.

In the case of weak correlations, either for positive correlations (Figure 3) or negative correlations (Annex 4), the generalized chi-square and the screening approaches were the most powerful ones. However, the power of the screening approach decreased when the number of parasites was high (>7). The other approaches had very poor power for two parasites (<0.1). Compared with the network models, GLM had a poor power when the number of parasites exceeded four parasites.

**Figure 4:**
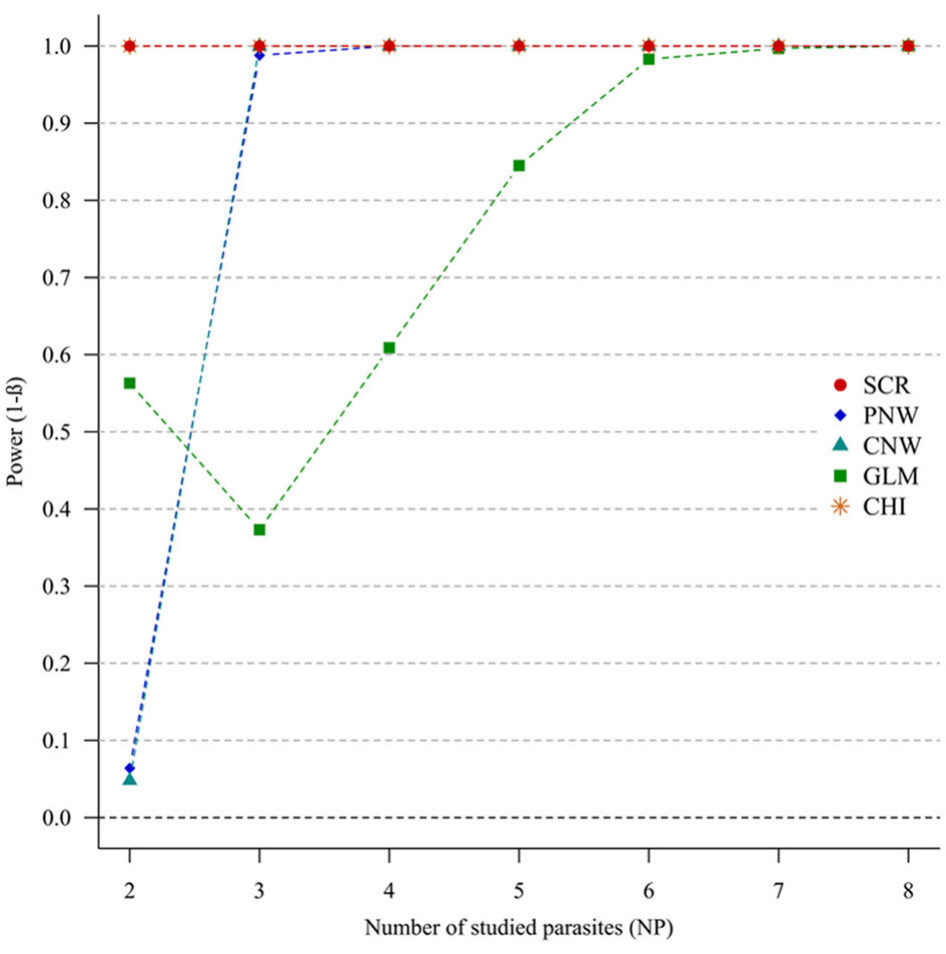
**Simulation outputs relative to the test of power (1-β) for the four approaches used to detect parasite structuring, with strong positive correlations**. CHI, generalized chi-square test; PNW, parasite network model; CNW, combination network model; GLM, multinomial GLM model and SCR, the association screening approach. The number of parasites varied from two to eight and the size of the total host population was fixed to 1000.

For strong parasite correlations, either for the positive correlation study (Figure 4) or the negative correlation study (Annex 5), all approaches had a good power (>0.7), except the parasite network for two parasites (<0.06).

Finally, the same trends were observed for smaller host population size (NH = 500). Power estimates were moderately lower (for weak correlations see Annexes 6A and 6B and for strong correlations see Annexes 7A and 7B).

### Case studies: association studies in two populations infected by multiple parasites

#### Microtus agrestis population with multi-infected hosts

All approaches, except the parasite network model (observed connectance: 0.197, *p*-value: 0.530), revealed a significant overall parasite association within the 887 field voles: observed connectance of host individuals was equal to 0.170 and significant (*p*-value: 0.034) for the combination network, observed residual deviance was equal to 0.179 and significant (*p*-value: 0.002) for the GLM, observed generalized chi-square was equal to 111.08 with 16 degrees of freedom and significant (*p*-value < 0.001) and also for the association screening model (*p*-value < 0.001).

Five parasite combinations were significant when subjected to the screening analysis, among which only one depicted parasite association: the association of *B. doshiae* and *Babesia microti* was underrepresented compared to expected (*p*-value < 0.001). The four other combinations were combinations where host individuals were infected by one and only one parasite. Infections by *B. doshiae* (*p*-value < 0.001), *B. grahamii* (*p*-value < 0.001) and *Babesia microti* on their own were all over-represented compared to expected (*p*-value < 0.001). Finally, the number of non-infected individuals was underrepresented compared to expected (*p*-value < 0.001).

#### Bovine population with multi-infected hosts

All approaches, except the GLM model (observed residual deviance: 0.186, *p*-value: 0.776), revealed a significant overall parasite association within the 600 cattle: observed connectance of host individuals was equal to 0.716 and significant (*p*-value < 0.001) for the parasite network, observed connectance of host individuals was equal to 0.190 and significant (*p*-value < 0.001) for the combination network, observed generalized chi-square was equal to 196.94 with 8 degrees of freedom and significant (*p*-value < 0.001) and also for the association screening model (*p*-value < 0.001).

Four parasite combinations were found significant using the screening analysis, among which two depicted parasite associations: individuals infected by *T. parva*, *T. mutans* and *T. velifera*, were significantly over-represented (*p*-value < 0.001). Those infected by *T. parva* and *T. velifera* were significantly underrepresented (*p*-value < 0.001). Individuals infected by *T. velifera* were underrepresented compared to expected (*p*-value < 0.001). Finally, there were less non-infected individuals than expected (*p*-value < 0.001).

**Figure 5:**
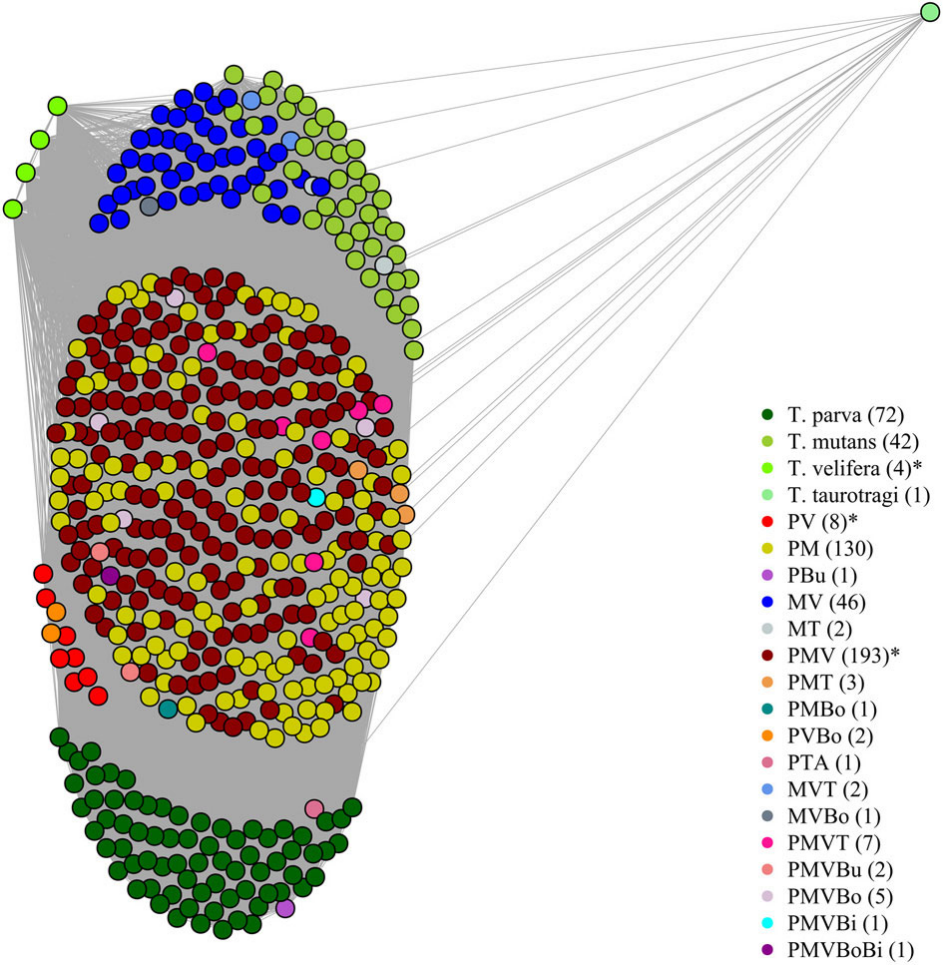
**Graphical representation of the structuring of host, using parasite network approach, for the dataset described in Salih et al. (2007)**. *Theileria parva* (P), *T. mutans* (M), *T. velifera* (V), *T. taurotragi* (T), *T. buffeli* (B), *T. annulata* (A), *Babesia bovis* (Bo) and *B. bigemina* (Bi). Each dot represent a host individual. Each color represent a parasite combination and the number of individual per combination is shown in brackets. Non-infected individuals were not shown. The asterisks highlight the significant combinations, which were found using the association screening approach.

**Figure 6:**
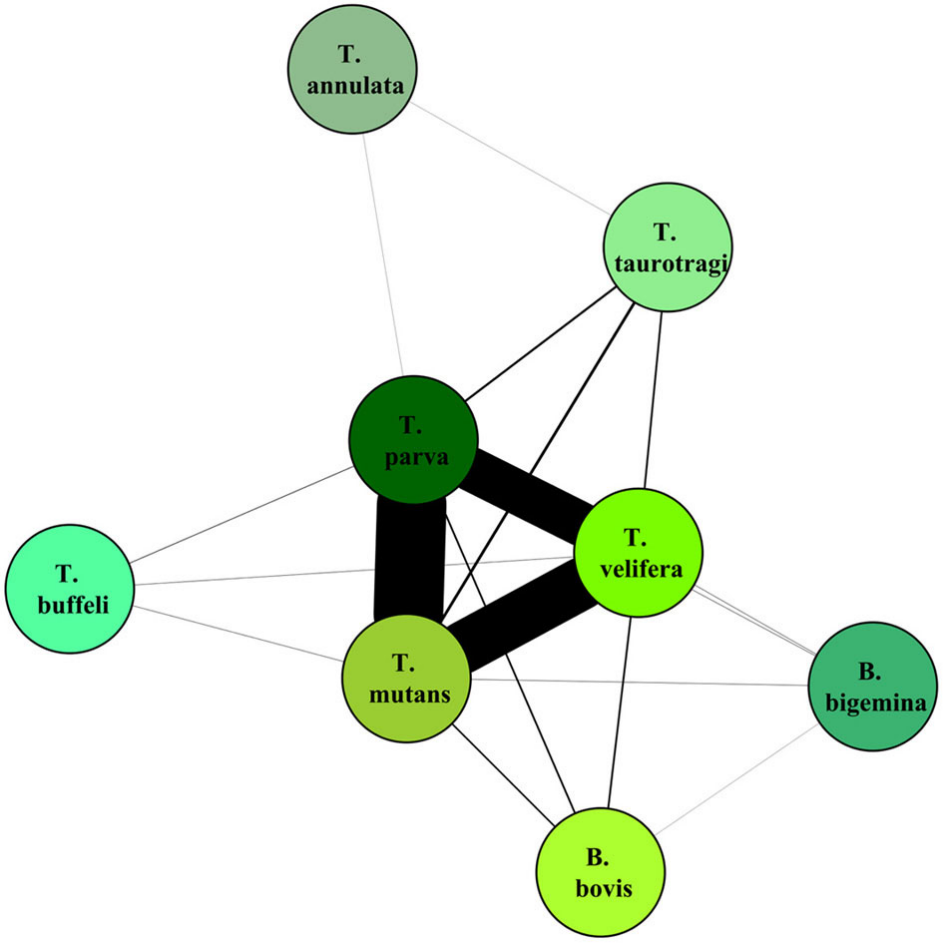
**Graphical representation of the structuring of parasite, using parasite network approach, for the dataset described in Salih et al. (2007)**. The lines width is proportional to the number of individual hosts involved.

On the graphical representations of the structuring of hosts we noted that individual hosts were divided in six main groups, two of which were associations detected with the screening approach (Figure 5). Graphical representations of the structuring of parasites highlight the three most prevalent parasite which were significant with the screening approach (Figure 6).

## Discussion

In the age of genomics and the advent of new technologies (e.g., high-throughput sequencing), it has become possible to identify intra-host parasite communities (e.g., Cheval et al., 2011; Vayssier-Taussat et al., 2013). Being able to statistically identify parasites that are associated is a considerable asset to speed up research focusing on the mechanisms driving interactions between parasites. Here we proposed a new powerful approach (“association screening”) to test the overall presence of parasite associations and identify the parasite combinations that are significantly over- or under-represented. The screening approach and the generalized chi-square test appeared to be the most suitable models to detect the presence of parasite associations. The main advantage of the screening approach is that significant parasite combinations can be statistically identified, in addition to knowing whether parasites are overall associated. To our knowledge, this is the only approach identifying precise parasite combinations in the case of multiple infections in cross-sectional studies.

Both, the parasite screening and the generalized chi-square approaches were also very powerful to detect parasite associations due to the presence of subpopulations. Thus, the differentiation between associations, which are due to confounding factors (which structure host populations into subpopulations) and parasite interactions was not possible. This differentiation was also not possible with the multinomial GLM and network models. To counter this problem, if the influence of a factor in the observed associations is suspected, this factor should be taken into account. For screening model, network approaches and generalized chi-square model, the dataset should be divided into different subpopulations according to the factor levels. The presence of parasite associations should then be tested within each subpopulation. The drawback of this strategy is that multiple statistical tests are used, increasing the alpha risk (Agresti, 2002). Contrary to these approaches the impact of potential confounding factors can be directly tested using a GLM model. However, it is also the least powerful of the approaches tested to identify structures of host and parasite populations. In any case, factors can only be tested when they are measured (e.g., Bajer et al., 2001; Pawelczyk et al., 2004). These results highlight the limits of exploratory approaches, emphasizing that conclusions should be further tested by other approaches such as longitudinal or experimental studies. For instance, for intestinal viruses that were found to be more prevalent in the presence of particular intestinal bacteria (Kuss et al., 2011; Pennisi, 2011), further experimental studies both confirmed this result and identified the mechanisms driving this interaction (Kane et al., 2011).

In the last few years, the use of network analysis has grown impressively (Bascompte, 2007; Poisot et al., 2012) as they give attractive graphical representations, can be used on big datasets and mimic ecological pathways (e.g., parasite transmission in Godfrey, 2013). Network analyses have been used in community ecology studies of interacting species (e.g., pollinators, parasites, Poulin, 2010) or to identify “hub” individuals, which contribute the most to parasite spread (for instance in a lizard population in Fenner et al., 2011). Therefore, within a given population, links between vertices were randomly generated to identify vertices that have more or less connection than expected. Here, we worked on networks with two types of populations (hosts and parasites). We randomly generated links between the two populations, i.e., parasites or combinations and hosts. Two hosts were associated when they had either at least one parasite in common (for the parasite network) or a unique similar combination (because the different combinations are exclusive). We found that both network approaches had rather good power, although less than the generalized chi-square and screening approaches, suggesting the need to explore more deeply some proprieties of networks (such as modularity) linked to parasite co-occurrences. Moreover, networks remain interesting for graphic representation of links between both parasite and hosts (e.g., Boutin et al., 2013).

Increasing the population size of hosts enhanced, as expected, the power of all tests. However, in real life, one should make sure that the number of hosts is at least greater than the number of combinations. Regarding the generalized chi-square approach, the main drawback is the requirement of a minimum number of five individuals for each possible combination of infections (Agresti, 2002), forcing that include less than five individuals to be merged. This loss of information can potentially mask putative association signals. Similarly, the multinomial GLM removes combinations without any hosts. In doing so, the number of degrees of freedom is decreased. This explains the low power of the multinomial GLM compared to the other approaches. For the generalized chi-square, as the combinations with less than 5 hosts were pooled, this maintains a certain stability in the sample size, and therefore, limits the power loss. The strength of host structuring, i.e., differences in prevalence and sample sizes between subpopulations, had an impact on the ability of approaches to detect a structure. The more the subpopulations were balanced in sample size, the better the structure of hosts could be identified. For both host structured and parasite structured simulations, we observed that there was generally a maximum of power in relation to the number of parasites. Indeed, the power first increased with the number of parasites. This is because the number of combinations also increases, and, therefore, there is more available information to discriminate between groups of individuals. For some approaches, at high numbers of parasites, the power decreased. This is due to the fact that the number of combinations is too high compared the number of hosts. In addition, we found that the power was better when parasites were strongly associated.

The α risk (or the type one error) was tightly controlled for all approaches, i.e., all approaches minimized the risk of concluding that the presence of associations was significant when in fact the presence of associations was random. This was true even for increased numbers of studied parasites (Figure 1). The association screening model, with an α risk of 0.04%, was a more conservative test than the others approaches. So when the screening approach detects structure in the host population, there is a strong chance that it is real.

The four approaches presented here can be conducted without any *a priori* biological knowledge. Thus, it is important to interpret results in light of current knowledge of the biology of studied parasites. It is also critical to remember that there is a risk in using all these four methods on the same dataset (i.e., problem of multiple tests, Bland and Altman, 1995; Agresti, 2002). Ideally, only one method should be used. If the aim of the analysis is to investigate in the first instance the associations, and then to go further in a second step (e.g., interaction study), the association screening approach is the most suitable.

Concerning the *M. agrestis* dataset, a significant parasite association was detected by all approaches except the parasite network. The screening approach found that *B. doshiae* (15.4%) and *B. microti* (34.4%) were negatively associated. Thus, we were examining a case of six studied parasites with a negative correlation. In the power study of strong negative correlation for six parasites, all approaches had a good power (Annex 5). The network approach may not have been significant in the field study due to the fact that our power study examined only correlations between parasites at 10 and 60% prevalence. The negative association between *B. doshiae* and *B. microti* corroborates previous findings from analyses of a longitudinal dataset from the same study system of Telfer et al. (2010) and Sherlock et al. (2013). Telfer et al. (2010) found that *B. microti* was negatively associated with *Bartonella* spp. and *vice versa*. Unlike here, they also detected that *A. phagocytophilum* was associated with *Bartonella* spp. and *B. microti*. In a Bayesian analysis of a smaller subset of the longitudinal dataset that had information on *Bartonella* species identity, Sherlock et al. (2013) found negative associations between *B. microti* and the three *Bartonella* species, *B. doshiae*, *B. taylorii*, and *B. grahamii*. The longitudinal nature of the datasets allowed these studies to analyse the impact of current and previous exposure (Telfer et al., 2010), or simply previous exposure (Sherlock et al., 2013), on the probability of infection and, in the case of Sherlock et al. (2013), the probabilities of recovery and reinfection. Clearly, this is not possible with a simple model of associations as in the screening model or with the three other studied approaches. In addition, both the longitudinal studies considered more individuals and captures (>5900 individuals and >14000 captures for Telfer et al., 2010; >1800 individuals and >4300 captures for Sherlock et al., 2013), and used GLM approaches to account for potentially confounding variables such as weight, sex and season. As infection probabilities for all the parasites included in this dataset have been shown to vary by such variables (Telfer et al., 2007, 2010), structuring of the host population may mask or enhance the associations identified using the cross-sectional approaches considered in this study and this, or increases sample size, could explain the observed differences between the current study and their findings. This highlights the need to consider confounding variables prior to conducting association analyses for hosts from wild populations. The mechanism behind the antagonistic interaction between *B. microti* and *Bartonella* spp. is unknown, but could be related to competition for host resources (both parasites infect erythrocytes) or cross-effective immune responses.

For the bovine dataset (Salih et al., 2007), all approaches except the GLM model, identified a significant parasite association in the bovine population. This seemed to be consistent with the power study, in the majority of cases studied, the GLM approach was the least powerful. The screening approach found that *T. parva*, *T. mutans*, and *T. velifera* were positively associated, whilst *T. parva* and *T. velifera*, were negatively associated. Apart from the fact that confounding factors are also unknown, to date the possible biological reasons for these associations can only be speculated on. Possible associations can be due to association found in vectors. Indeed *T. mutans* and *T. velifera* are both vectorised by *Amblyomma* spp. (Anonymous, 1983; Sugimoto and Fujisaki, 2002; Salih et al., 2008). Another explanation for significant associations could be that *T. parva*, which is pathogenic, would modify the host susceptibility for *T. mutans* and *T. velifera*, which are both benign infections (Uilenberg, 1981; Sugimoto and Fujisaki, 2002).

After identifying potential associations within a parasite community, the potential interactions between the parasites can be studied. A mechanistic model was developed to study more precisely the relationships between *B. microti*, *A. phagocytophilum*, and *Bartonella* sp. in *M. agrestis* (Sherlock et al., 2013). In the same way, it would be interesting to implement a mechanistic model to better understand the relationships between *Theileria* species in cattle.

One major advance would be to incorporate potentially confounding factors for the generalized chi-square and screening approaches. This has already been achieved for the chi-square test that tested two by two parasite association (Hellard et al., 2012). Hellard et al. have integrated confounding factors linked to the prevalence of parasites. Similarly, this could be implemented into the screening approach. Network analyses are also particularly promising for studying and representing parasite association, although more investigations are needed using both real and simulated data (Poisot et al., 2012, 2013).

## Author contributions

Analyzed the data: Elise Vaumourin and Patrick Gasqui. Contributed analysis tools: Patrick Gasqui and Elise Vaumourin. Wrote the paper: Elise Vaumourin, Patrick Gasqui, Gwenaël Vourc'h, Muriel Vayssier-Taussat, Sandra Telfer, Serge Morand, and Nathalie Charbonnel. Supervised the work: Patrick Gasqui, Gwenaël Vourc'h, and Muriel Vayssier-Taussat. Designed and perform the field work: Sandra Telfer, Xavier Lambin, Diaeldin Salih, and Ulrike Seitzer.

### Conflict of interest statement

The authors declare that the research was conducted in the absence of any commercial or financial relationships that could be construed as a potential conflict of interest.
