# Supplementary material for: To be or not to be associated: power study of four statistical modeling approaches to identify parasite associations in cross-sectional studies
Source: Front Cell Infect Microbiol. 2014 May 15;4:62. doi: 10.3389/fcimb.2014.00062 (PMC4030204; doi:10.3389/fcimb.2014.00062)
Supplement: Supplementary file 1 [file DataSheet1.PDF]

# *Supplementary Material*

# To Be or Not to Be Associated: Power study of four statistical modeling approaches to identify parasite associations in cross-sectional studies

**Elise Vaumourin[1,2*], Gwenaël Vourc'h[1], Sandra Telfer[3], Xavier Lambin[3], Diaeldin Salih[4], Ulrike Seitzer[5], Serge Morand[6], Nathalie Charbonnel[7], Muriel Vayssier-Taussat[2], Patrick Gasqui[1]**

[1]UR346 Epidémiologie Animale, INRA, Saint Genès Champanelle, France
[2]USC BIPAR, INRA-Anses-ENVA, Maisons-Alfort, France
[3]School of Biological Sciences, University of Aberdeen, Aberdeen, UK
[4]Veterinary Research Institute, Khartoum, Sudan
[5]Division of Veterinary Infection Biology and Immunology, Borstel, Germany
[6]Institut des Sciences de l'Evolution (CNRS /IRD / UM2), University of Montpellier 2, Montpellier, France
& Animal et Gestion Intégrée des Risques, CIRAD, Montpellier, France
[7]UMR CBGP (INRA ⁄ IRD ⁄ Cirad ⁄ Montpellier SupAgro), INRA, Montpellier, France

*** Correspondence:** Elise Vaumourin, UR346 Epidémiologie Animale, INRA, Centre de recherche de Clermont-Ferrand – Theix, Saint Genès Champanelle, 63122, France.
elise.vaumourin@clermont.inra.fr

**Annex 1 Simulation outputs relative to the test of power (1-β) for the four approaches, with a prevalence ratio equal to 1.5.** CHI: generalised chi-square test; PNW: parasite network model; CNW: combination network model; GLM: multinomial GLM model and SCR: the association screening approach. The number of parasites varied from two to eight, the size of the total host population was fixed to 1000 and the ratios of host subpopulation sizes varied **(A)** 0.2. **(B)** 0.3. **(C)** 0.4. **(D)** 0.5.

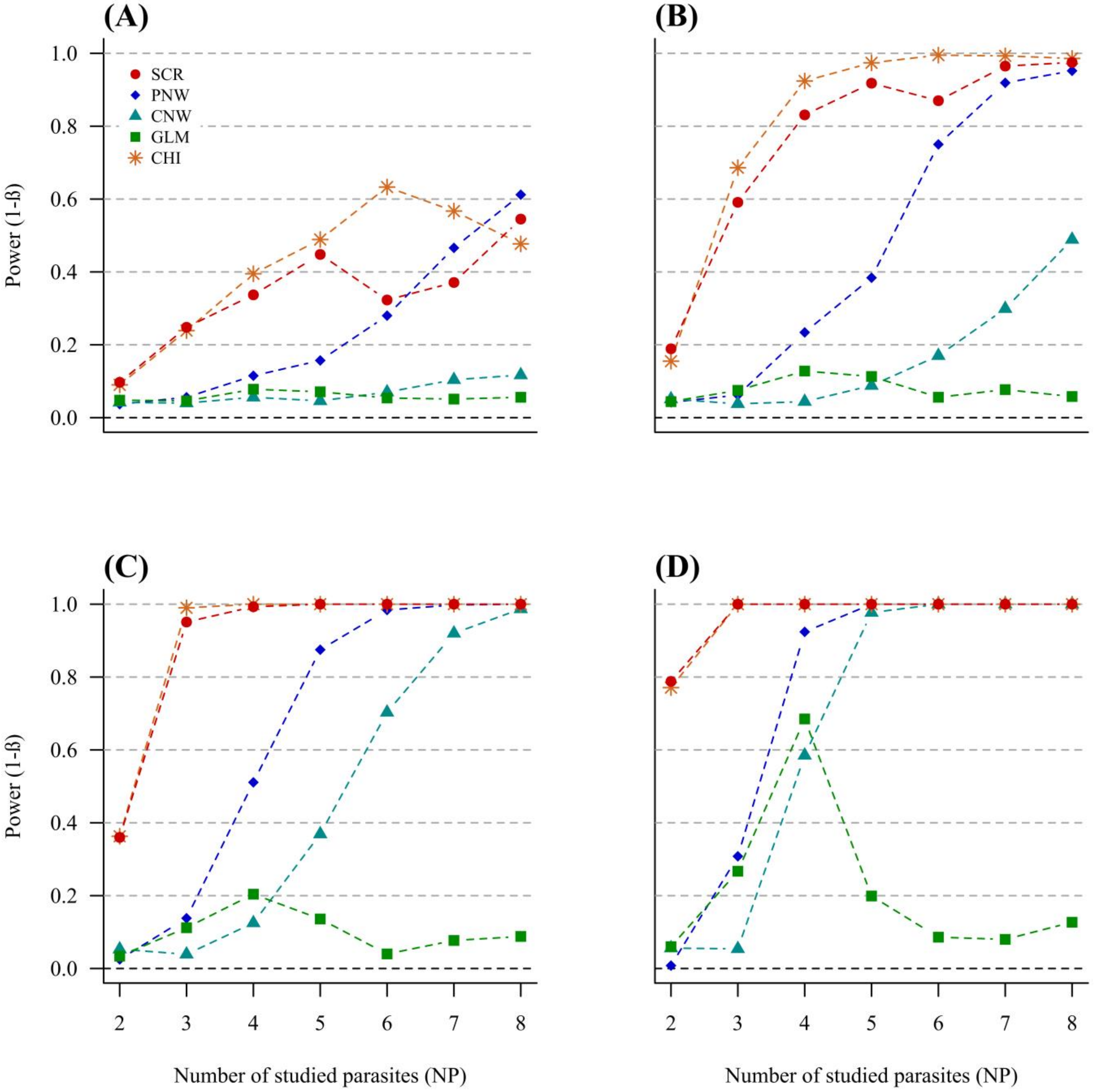

**Annex 2 Simulation outputs relative to the test of power (1-β) for the four approaches, with a prevalence ratio equal to 0.5.** CHI: generalised chi-square test; PNW: parasite network model; CNW: combination network model; GLM: multinomial GLM model and SCR: the association screening approach. The number of parasites varied from two to eight, the size of the total host population was fixed to 500 and the ratios of host subpopulation sizes varied **(A)** 0.2. **(B)** 0.3. **(C)** 0.4. **(D)** 0.5.

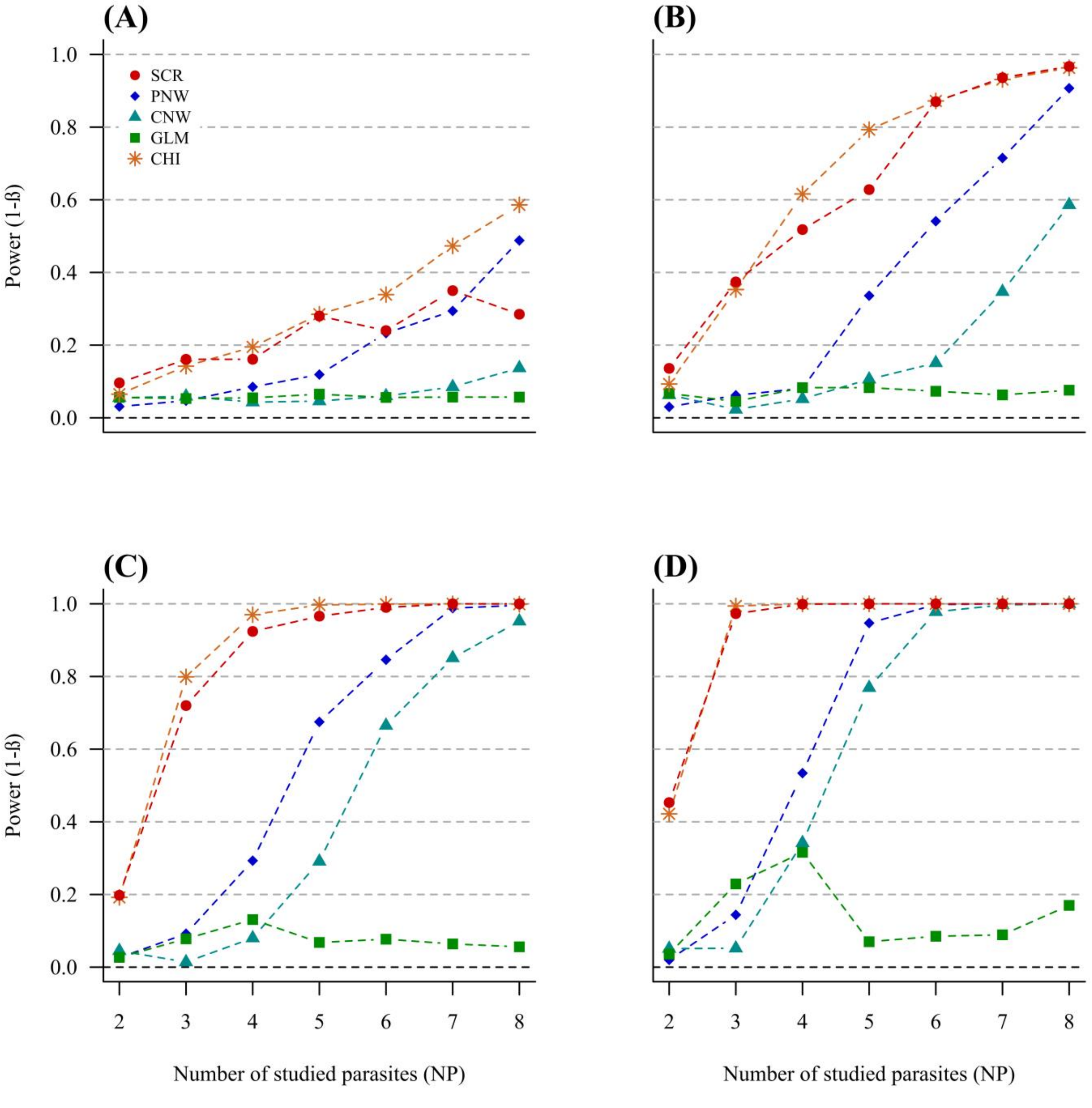

**Annex 3 Simulation outputs relative to the test of power (1-β) for the four approaches, with a prevalence ratio equal to 1.5.** CHI: generalised chi-square test; PNW: parasite network model; CNW: combination network model; GLM: multinomial GLM model and SCR: the association screening approach. The number of parasites varied from two to eight, the size of the total host population was fixed to 500 and the ratios of host subpopulation sizes varied **(A)** 0.2. **(B)** 0.3. **(C)** 0.4. **(D)** 0.5.

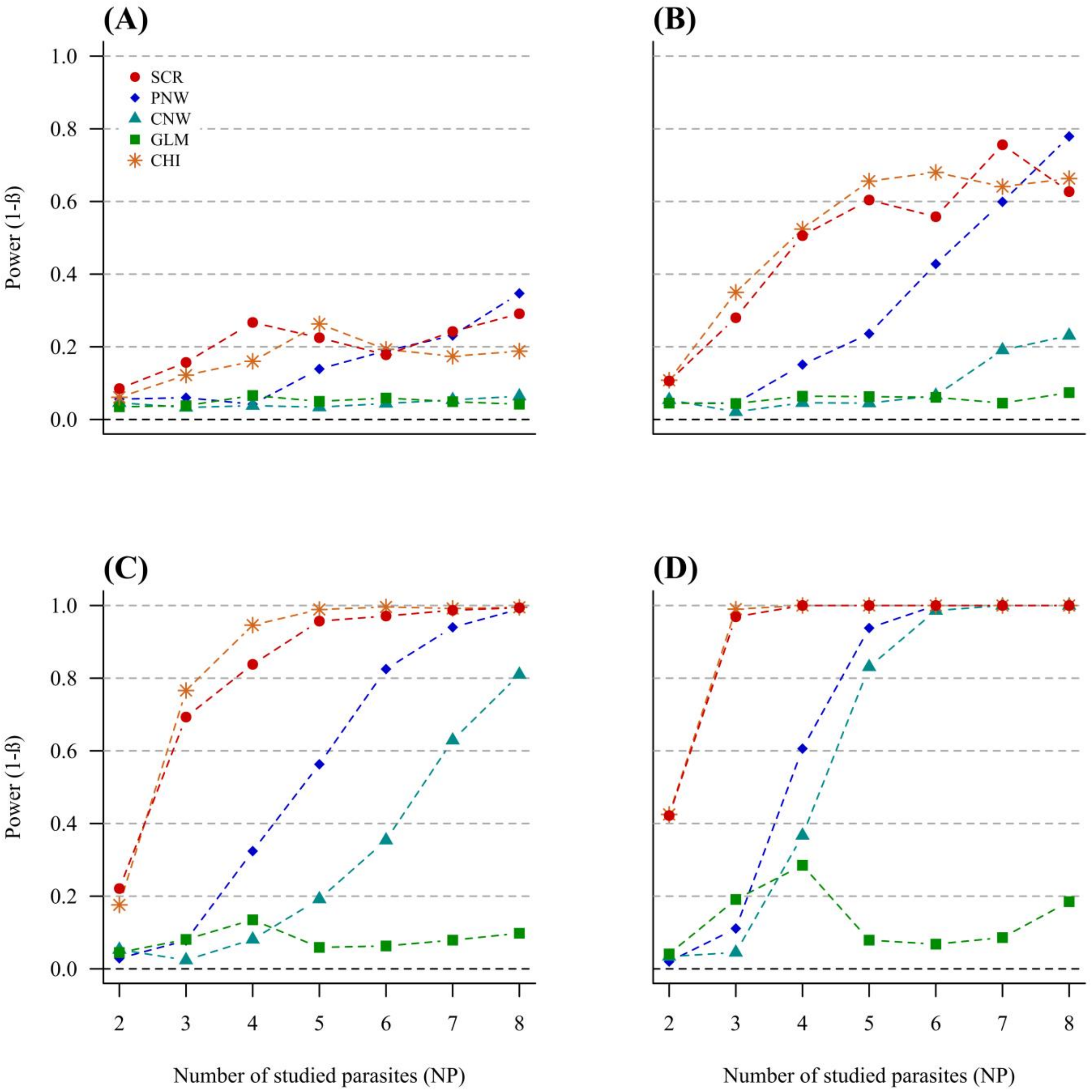

**Annex 4 Simulation outputs relative to the test of power (1-β) for the four approaches used to detect parasite structuring, with weak negative correlations.** CHI: generalised chi-square test; PNW: parasite network model; CNW: combination network model; GLM: multinomial GLM model and SCR: the association screening approach. The number of parasites varied from two to eight and the size of the total host population was fixed to 1000.

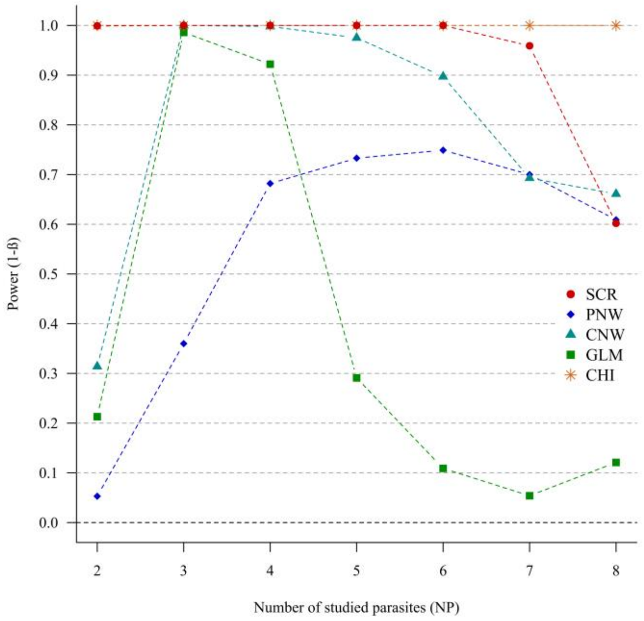

**Annex 5 Simulation outputs relative to the test of power (1-β) for the four approaches used to detect parasite structuring, with strong negative correlations.** CHI: generalised chi-square test; PNW: parasite network model; CNW: combination network model; GLM: multinomial GLM model and SCR: the association screening approach. The number of parasites varied from two to eight and the size of the total host population was fixed to 1000.

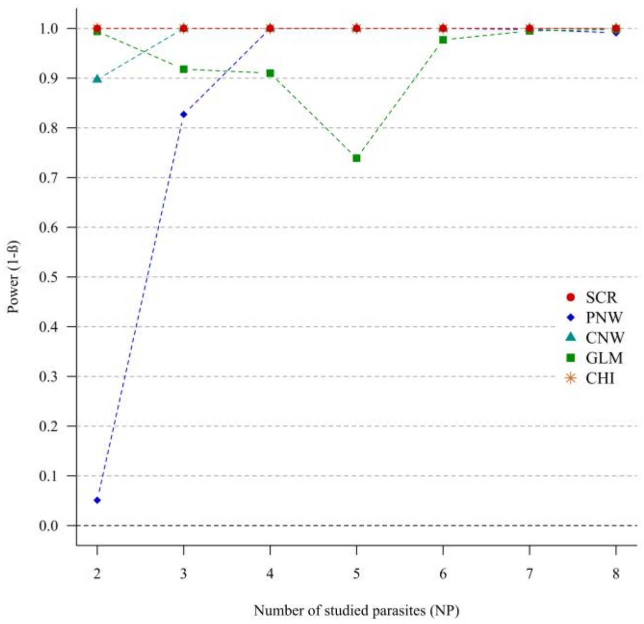

**Annex 6 Simulation outputs relative to the test of power (1-β) for the four approaches used to detect parasite structuring, with weak correlations.** CHI: generalised chi-square test; PNW: parasite network model; CNW: combination network model; GLM: multinomial GLM model and SCR: the association screening approach. The number of parasites varied from two to eight and the size of the total host population was fixed to 500. **(A)** Representation of negative correlations. **(B)** Representation of positive correlations.

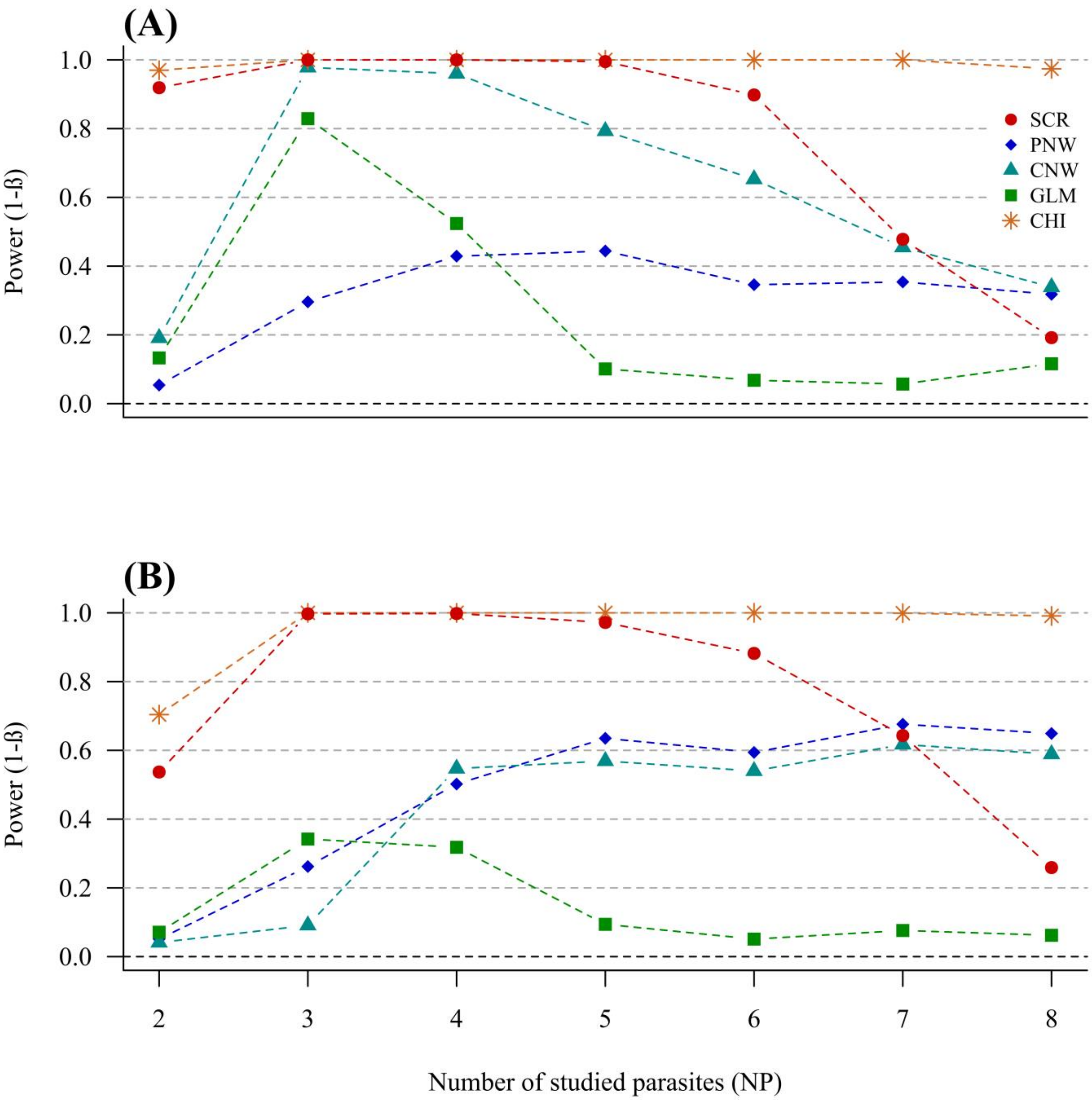

**Annex 7 Simulation outputs relative to the test of power (1-β) for the four approaches used to detect parasite structuring, with strong correlations.** CHI: generalised chi-square test; PNW: parasite network model; CNW: combination network model; GLM: multinomial GLM model and SCR: the association screening approach. The number of parasites varied from two to eight and the size of the total host population was fixed to 500. **(A)** Representation of negative correlations. **(B)** Representation of positive correlations.

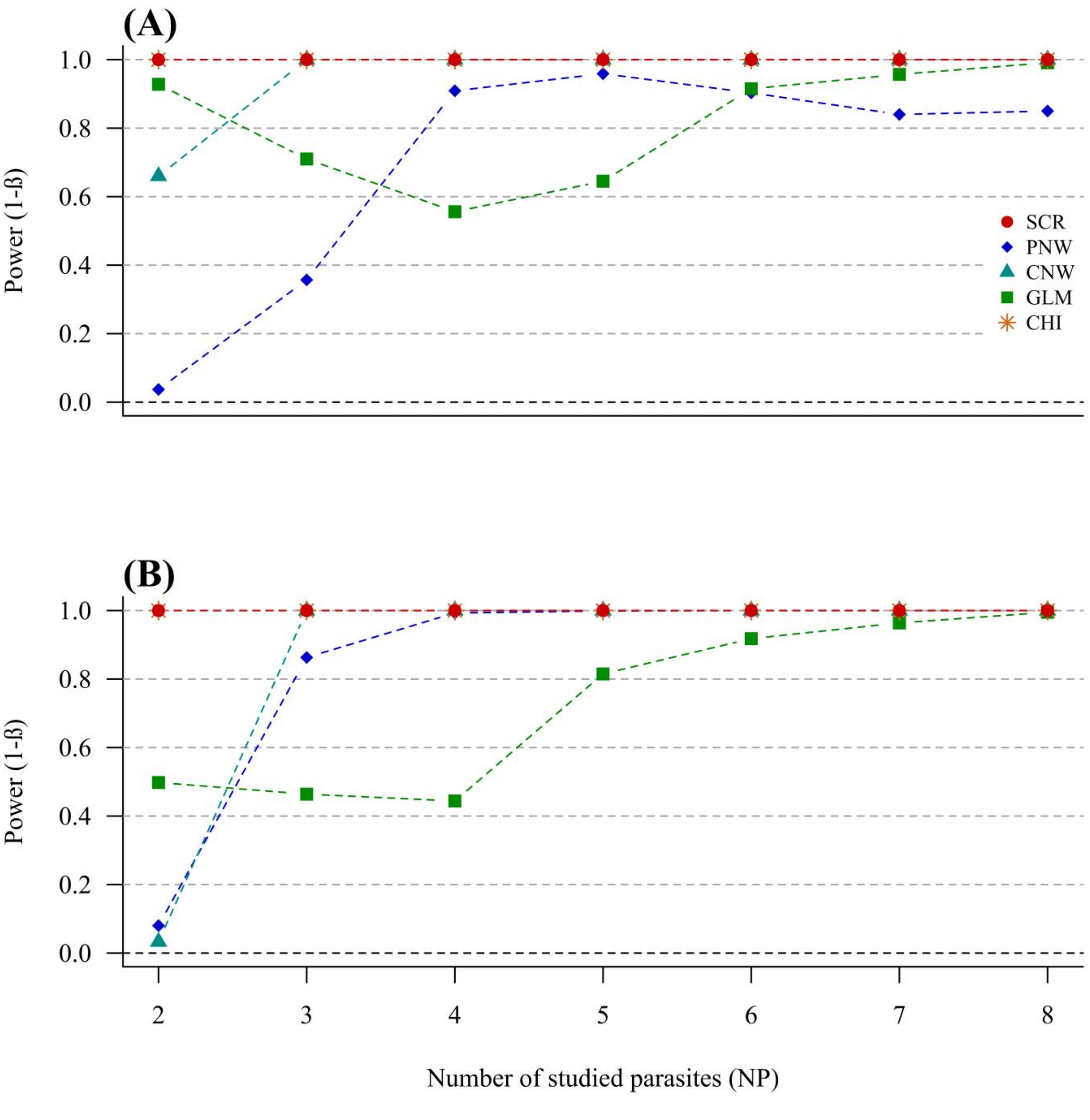

## Script 1 Function to apply the association screening approach.

```
#
library(boot)
#
FctTestScreenENV <- function(tabx) {
  xNS    <- 5000
  xNH    <- dim(tabx)[[1]]
  xNP    <- dim(tabx)[[2]]
  xpv <- apply(tabx,2,sum)/xNH
  xNC    <- 2**xNP
# Data structuring for a multinomial with NC combinations
  DatXMul <- array(rep(0,xNC*xNP),c(xNC,xNP))
  for ( k in 1:xNP ) {
    k1 <- 2**(xNP-k)
    k2 <- 2**(k-1)
    DatXMul[,k] <- rep( c(rep(1,k1),rep(0,k1)) ,k2) }
  #
  DatFMul <- rep(0,xNC)
  for ( k in 1:xNC ) {
    x <- 0
    for ( j in 1:xNP ) {
      if ( DatXMul[k,j] == 1 ) {
        x <- x+(10**(xNP-j)) } }
    DatFMul[k] <- x }
# Calculation of prevalence of combinations under H0
  xpvcomb <- rep(0.0,xNC)
  for ( i in 1:xNC ) {
    x <- 1.0
    for (  j in 1:xNP ) {
      if ( DatXMul[i,j] == 1 ) {
        x <- x*(xpv[j]) } else {
        x <- x*(1.0-xpv[j]) } }
    xpvcomb[i] <- x }
# Calculation of theoretical sample sizes for each combination
  xeffEstcomb <- xpvcomb*xNH
# Calculation of observed sample size for each combination
  taby <- array(rep(0,xNH*xNC),c(xNH,xNC))
  for ( i in 1:xNH ) {
    x <- 0
    for ( j in 1:xNP ) {
      if ( tabx[i,j] == 1 ) {
        x <- x+(10**(xNP-j)) } }
    z <- (1:xNC)[(DatFMul==x)]
    taby[i,z] <- 1 }
  xeffObscomb <- apply(taby,2,sum)
# Statistic of the screening under H0
  xresCountH0Qinf <- rep(0.0,xNC)
  xresCountH0Qsup <- rep(0.0,xNC)
  xresCountH0 <- array(rep(0.0,xNC*xNS),c(xNC,xNS))
 # Simulation of kth dataset
  for ( k in 1:xNS ) {
    xdata <- t(rmultinom(xNH, 1, prob=xpvcomb))
    # Recuperation of sample sizes for each combination
    xresCountH0[,k] <- apply(xdata,2,sum) }
# Calculation of 95% CI of the statistic under H0
# [ Confidence Envelopes for Curves ]
  ConfianceGlobal <- 0.95
  resenv <- envelope(mat = t(xresCountH0), level =
ConfianceGlobal)
  xresCountH0Qsup <- resenv$overall[1,]
  xresCountH0Qinf <- resenv$overall[2,]
# Calculation of P-value
##################################################
# WARNING : We work with discrete data (i.e. many values
are identical) so the calculation of the p-value should be used
with caution
#################################################
xresScr <- rep(0,xNC)
  # xresPvalue <- rep(0,xNC)                # WARNING
  # minPvalue0 <- 1.00                      # WARNING
  # minPvalue1 <- 0.05                      # WARNING
  for ( i in 1:xNC ) {
    xobs <- xeffObscomb[i]
    x <- xresCountH0[i,]
    if ( (xobs > xresCountH0Qsup[i])  ) {
      xresScr[i] <- 1
      # xresPvalue[i] <- length(x[(x>=xobs)])*2.0/xNS
                                            # WARNING
      # if ( xresPvalue[i] < minPvalue1 ) { minPvalue1 <-
xresPvalue[i] }                             # WARNING
      }
    if ( (xobs < xresCountH0Qinf[i])  ) {
      xresScr[i] <- 1
      # xresPvalue[i] <- length(x[(x<=xobs)])*2.0/xNS
                                            # WARNING
      # if ( xresPvalue[i] < minPvalue1 ) { minPvalue1 <-
xresPvalue[i] }                             # WARNING
      }
    # if ( xresScr[i] == 0 ) {              # WARNING
      # if ( xobs < median(x) ) {           # WARNING
        # xresPvalue[i] <- length(x[(x<=xobs)])*2.0/xNS
                                             # WARNING
        # if ( xresPvalue[i] > 1.0 ) xresPvalue[i] <-
xresPvalue[i]/2.0                           # WARNING
        # if ( xresPvalue[i] < minPvalue0 ) { minPvalue0 <-
xresPvalue[i] }                             # WARNING
        # } else {                          # WARNING
        # xresPvalue[i] <- length(x[(x>=xobs)])*2.0/xNS
                                            # WARNING
        # if ( xresPvalue[i] > 1.0 ) xresPvalue[i] <-
xresPvalue[i]/2.0                           # WARNING
        # if ( xresPvalue[i] < minPvalue0 ) { minPvalue0 <-
xresPvalue[i] }                             # WARNING
        # } }                               # WARNING
    }
  resScr <- sum(xresScr)
  if ( resScr != 0 ) {
    resTest <- list(resScr,
            # minPvalue1,                   # WARNING
            DatFMul,xresScr,
            # xresPvalue,                   # WARNING
            xeffObscomb,xresCountH0Qsup,xresCountH0Qinf)
} else {
    resTest <- list(resScr,
            # minPvalue0,                   # WARNING
            DatFMul,xresScr,
            # xresPvalue,                   # WARNING
            xeffObscomb,xresCountH0Qsup,xresCountH0Qinf)
}
  resTest
  }
```

## Script 2 Function to apply the generalized chi-square approach.

```
#
FctTestCHI2 <- function(tabx) {
  xNS    <- 5000
  xNH    <- dim(tabx)[[1]]
  xNP    <- dim(tabx)[[2]]
  xpv <- apply(tabx,2,sum)/xNH
  xNC    <- 2**xNP
# Data structuring for a multinomial with NC combinations
  DatXMul <- array(rep(0,xNC*xNP),c(xNC,xNP))
  for ( k in 1:xNP ) {
    k1 <- 2**(xNP-k)
    k2 <- 2**(k-1)
    DatXMul[,k] <- rep( c(rep(1,k1),rep(0,k1)) ,k2) }
  #
  DatFMul <- rep(0,xNC)
  for ( k in 1:xNC ) {
    x <- 0
    for ( j in 1:xNP ) {
      if ( DatXMul[k,j] == 1 ) { x <- x+(10**(xNP-j)) } }
    DatFMul[k] <- x }
# Calculation of prevalence of combinations under H0
  xpvcomb <- rep(0.0,xNC)
  for ( i in 1:xNC ) {
    x <- 1.0
    for (  j in 1:xNP ) {
      if ( DatXMul[i,j] == 1 ) {
        x <- x*(xpv[j]) } else {
        x <- x*(1.0-xpv[j]) } }
    xpvcomb[i] <- x }
# Calculation of theoretical sample sizes for each combination
  xeffEstcomb <- xpvcomb*xNH
  selkc <- ( xeffEstcomb > 5.0 )
  if ( sum(selkc) < xNC ) {
    selknc <- !selkc
    xNCmod <- sum(selkc) + 1 } else {
    selknc <- rep(0,xNC)
    xNCmod <- sum(selkc)  }
# Calculation of observed sample size for each combination
  taby <- array(rep(0,xNH*xNC),c(xNH,xNC))
  for ( i in 1:xNH ) {
    x <- 0
    for ( j in 1:xNP ) {
      if ( tabx[i,j] == 1 ) {
        x <- x+(10**(xNP-j)) } }
    z <- (1:xNC)[(DatFMul==x)]
    taby[i,z] <- 1 }
  xeffObscomb <- apply(taby,2,sum)
# Statistics of the generalized chi-square
  xdifeff <- (xeffObscomb-xeffEstcomb)
  xchi2   <- sum((xdifeff**2)/xeffEstcomb)
  ychi2   <- ((xdifeff**2)/xeffEstcomb)
  xdnc <- (sum(xeffObscomb[selknc])-sum(xeffEstcomb[selknc]))**2
  xenc <- sum(xeffEstcomb[selknc])
  if ( sum(selkc) < xNC ) {
    xchi2mod   <- sum((xdifeff[selkc]**2)/xeffEstcomb[selkc]) + (xdnc/xenc)
  } else { xchi2mod   <- sum((xdifeff**2)/xeffEstcomb) }
# Calculation of P-value and contribution
  xresCHI2mod <- 1.0-pchisq(xchi2mod,(xNCmod-1))
  alphaIndiv <- (0.05)
  x <- ychi2/xchi2
  selx <- (x>(xchi2mod/xNC))
  resTest <-list((xresCHI2mod<alphaIndiv),xresCHI2mod,xchi2mod,(xNCmod-1),DatFMul[selx],x[selx])
  resTest  }
```

## Script 3 Function to apply the multinomial GLM approach.

```
#
FctTestGLM <- function(tabx) {
  xNS    <- 1000
  xNH    <- dim(tabx)[[1]]
  xNP    <- dim(tabx)[[2]]
  xpv <- apply(tabx,2,sum)/xNH
  xNC    <- 2**xNP
# Data structuring for a multinomial with NC combinations
  DatXMul <- array(rep(0,xNC*xNP),c(xNC,xNP))
  for ( k in 1:xNP ) {
    k1 <- 2**(xNP-k)
    k2 <- 2**(k-1)
    DatXMul[,k] <- rep( c(rep(1,k1),rep(0,k1)) ,k2) }
  #
  DatFMul <- rep(0,xNC)
  for ( k in 1:xNC ) {
    x <- 0
    for ( j in 1:xNP ) {
      if ( DatXMul[k,j] == 1 ) {
        x <- x+(10**(xNP-j)) } }
    DatFMul[k] <- x }
# Calculation of prevalence of combinations under H0
  xpvcomb <- rep(0.0,xNC)
  for ( i in 1:xNC ) {
    x <- 1.0
    for (  j in 1:xNP ) {
      if ( DatXMul[i,j] == 1 ) {
        x <- x*(xpv[j]) } else {
        x <- x*(1.0-xpv[j]) } }
    xpvcomb[i] <- x }
# Calculation of theoretical sample sizes for each combination
  xeffEstcomb <- xpvcomb*xNH
# Calculation of observed sample size for each combination
  taby <- array(rep(0,xNH*xNC),c(xNH,xNC))
  for ( i in 1:xNH ) {
    x <- 0
    for ( j in 1:xNP ) {
      if ( tabx[i,j] == 1 ) {
        x <- x+(10**(xNP-j)) } }
    z <- (1:xNC)[(DatFMul==x)]
    taby[i,z] <- 1 }
  xeffObscomb <- apply(taby,2,sum)
# Statistic of the multinomial GLM under H0
  xresVraisH0 <- rep(0.0,xNS)
  xresVraisH0Qinf <- 0.0
  xresVraisH0Qsup <- 0.0
 # Simulation of kth dataset
  for ( k in 1:xNS ) {
    xdata <- t(rmultinom(xNH, 1, prob=xpvcomb))
    # Direct calculation of the statistics without factors
    vxds <- apply(xdata,2,sum)
    selk <- (vxds!=0.0)
    resMVO <- -2.0*sum(vxds[selk]*log(vxds[selk]/xNH))
    xNC0 <- xNC - sum(selk)
    resddl <- ((xNC-1)*(xNH-1)) - (xNC0*(xNH-1))
    xresVraisH0[k] <- resMVO/resddl }
# Calculation of 95% CI of the statistic under H0
  alphaIndiv <- (0.05)/2.0
  x <- quantile(xresVraisH0,probs=c(alphaIndiv,(1.0-
alphaIndiv)))
  xresVraisH0Qinf <- x[1]
  xresVraisH0Qsup <- x[2]
# Statistic of multinomial GLM without factors
  vxds <- apply(taby,2,sum)
  selk <- (vxds!=0.0)
  resMVO <- -2.0*sum(vxds[selk]*log(vxds[selk]/xNH))
  xNC0 <- xNC - sum(selk)
  resddl <- ((xNC-1)*(xNH-1)) - (xNC0*(xNH-1))
  xresVrais <- resMVO/resddl
# Calculation of P-value
  xresGLM <- 0
  xpval <- 1.0
  if ( (xresVrais > xresVraisH0Qsup)  ) {
    xresGLM <- 1
    xpval <-
length(xresVraisH0[(xresVraisH0>=xresVrais)])*2.0/xNS }
  if ( (xresVrais < xresVraisH0Qinf)  ) {
    xresGLM <- 1
    xpval <-
length(xresVraisH0[(xresVraisH0<=xresVrais)])*2.0/xNS }
  if ( xresGLM == 0 ) {
    if (xresVrais < median(xresVraisH0)) {
      xpval <-
length(xresVraisH0[(xresVraisH0<=xresVrais)])*2.0/xNS }
else {
      xpval <-
length(xresVraisH0[(xresVraisH0>=xresVrais)])*2.0/xNS } }
  resGLM <- c(xresGLM,xpval, xresVrais)
  }
```

## Script 4 Function to apply the parasite and combination network approach.

```
#
library(igraph)
#
FctTestNet <- function(tabx) {
  xNS    <- 1000
  xNH    <- dim(tabx)[[1]]
  xNP    <- dim(tabx)[[2]]
  xpv <- apply(tabx,2,sum)/xNH
  xNC    <- 2**xNP
# Data structuring for a multinomial with NC combinations
  DatXMul <- array(rep(0,xNC*xNP),c(xNC,xNP))
  for ( k in 1:xNP ) {
    k1 <- 2**(xNP-k)
    k2 <- 2**(k-1)
    DatXMul[,k] <- rep( c(rep(1,k1),rep(0,k1)) ,k2) }
  DatFMul <- rep(0,xNC)
  for ( k in 1:xNC ) {
    x <- 0
    for ( j in 1:xNP ) {  if ( DatXMul[k,j] == 1 ) {
        x <- x+(10**(xNP-j)) } }
    DatFMul[k] <- x }
# Calculation of prevalence of combinations under H0
  xpvcomb <- rep(0.0,xNC)
  for ( i in 1:xNC ) {
    x <- 1.0
    for (  j in 1:xNP ) {  if ( DatXMul[i,j] == 1 ) {
        x <- x*(xpv[j]) } else { x <- x*(1.0-xpv[j]) } }
    xpvcomb[i] <- x }
# Calculation of theoretical sample sizes for each combination
  xeffEstcomb <- xpvcomb*xNH
# Calculation of observed sample size for each combination
  taby <- array(rep(0,xNH*xNC),c(xNH,xNC))
  for ( i in 1:xNH ) {
    x <- 0
    for ( j in 1:xNP ) {  if ( tabx[i,j] == 1 ) {
        x <- x+(10**(xNP-j)) } }
    z <- (1:xNC)[(DatFMul==x)]
    taby[i,z] <- 1 }
  xeffObscomb <- apply(taby,2,sum)
# Statistic of the networks under HO
  xresDens1H0 <- rep(0.0,xNS)
  xresDens1H0Qinf <- 0.0
  xresDens1H0Qsup <- 0.0
  xresDens2H0 <- rep(0.0,xNS)
  xresDens2H0Qinf <- 0.0
  xresDens2H0Qsup <- 0.0
 # Simulation of kth dataset
  for ( k in 1:xNS ) {
    xdata <- array(rep(0,xNH*xNP),c(xNH,xNP))
    for ( j in 1:xNP ) {
      xdata[,j] <- rbinom(xNH,size=1,prob=xpv[j]) }
    ydata <- array(rep(0,xNH*xNC),c(xNH,xNC))
    for ( i in 1:xNH ) {
      x <- 0
      for ( j in 1:xNP ) {  if ( xdata[i,j] == 1 ) {
          x <- x+(10**(xNP-j)) } }
      DatFMul==x
      z <- (1:xNC)[(DatFMul==x)]
      ydata[i,z] <- 1 }
    # Calculation of the network connectance
    # 1 - matrix : NH*NP
    matSim <- as.matrix(xdata)
    graphSim <- graph.incidence(matSim)
    projecSim <- bipartite.projection(graphSim)
    xresDens1H0[k] <- graph.density(projecSim[[1]])
    # 2 - matrix : NH*NC
    matSim <- as.matrix(ydata)
    graphSim <- graph.incidence(matSim)
    projecSim <- bipartite.projection(graphSim)
    xresDens2H0[k] <- graph.density(projecSim[[1]]) }
# Calculation of 95% CI of the statistic under H0
  alphaIndiv <- (0.05)/2.0
  x <- quantile(xresDens1H0,probs=c(alphaIndiv,(1.0-
alphaIndiv)))
  xresDens1H0Qinf <- x[1]
  xresDens1H0Qsup <- x[2]
  x <- quantile(xresDens2H0,probs=c(alphaIndiv,(1.0-
alphaIndiv)))
  xresDens2H0Qinf <- x[1]
  xresDens2H0Qsup <- x[2]
# Calculation of the statistic for: 1 - matrix : NH*NP
  matSim <- as.matrix(tabx)
  graphSim <- graph.incidence(matSim)
  projecSim <- bipartite.projection(graphSim)
  xresDens1 <- graph.density(projecSim[[1]])
# Calculation of the statistic for: 2 - matrix : NH*NC
  matSim <- as.matrix(taby)
  graphSim <- graph.incidence(matSim)
  projecSim <- bipartite.projection(graphSim)
  xresDens2 <- graph.density(projecSim[[1]])
# Calculation of P-value
  xres1 <- 0
  xres2 <- 0
  xpval1 <- 1.0
  xpval2 <- 1.0
  if ( (xresDens1 > xresDens1H0Qsup)  ) {
    xres1 <- 1
    xpval1 <-
length(xresDens1H0[(xresDens1H0>=xresDens1)])*2.0/xNS}
  if ( (xresDens1 < xresDens1H0Qinf)  ) {
    xres1 <- 1
    xpval1 <-
length(xresDens1H0[(xresDens1H0<=xresDens1)])*2.0/xNS}
  if ( xres1 == 0 ) {
    if (xresDens1 < median(xresDens1H0)) {
      xpval1 <-
length(xresDens1H0[(xresDens1H0<=xresDens1)])*2.0/xNS
} else {      xpval1 <-
length(xresDens1H0[(xresDens1H0>=xresDens1)])*2.0/xNS
} }
  if ( (xresDens2 > xresDens2H0Qsup)  ) {  xres2 <- 1
    xpval2 <-
length(xresDens2H0[(xresDens2H0>=xresDens2)])*2.0/xNS}
  if ( (xresDens2 < xresDens2H0Qinf)  ) {
    xres2 <- 1
    xpval2 <-
length(xresDens2H0[(xresDens2H0<=xresDens2)])*2.0/xNS}
  if ( xres2 == 0 ) {
    if (xresDens2 < median(xresDens2H0)) {  xpval2 <-
length(xresDens2H0[(xresDens2H0<=xresDens2)])*2.0/xNS
} else {      xpval2 <-
length(xresDens2H0[(xresDens2H0>=xresDens2)])*2.0/xNS
} }
  resNet <- c( xres1, xpval1, xresDens1, xres2, xpval2,
xresDens2 )
  resNet }
```
